# Cinematic rendering in CT angiography: a pictorial review of clinical applications

**DOI:** 10.1186/s13244-026-02310-8

**Published:** 2026-05-21

**Authors:** Chong-ze Yang, Hua-song Cai, Lei Ding, Xiao-fang Chen, Ming-jie Chen, Li-na Chen, Ying-xia Yang, Zhen-peng Peng, Shi-Ting Feng

**Affiliations:** 1 Department of Radiology, The First Affiliated Hospital, Sun Yat-Sen University, Guangzhou, China; 2 Department of Radiology, Guangxi Hospital Division of the First Affiliated Hospital, Sun-Yat-Sen University, Nanning, China; 3 CT Collaboration, Siemens Healthineers Ltd., Shanghai, China

**Keywords:** Cinematic rendering, Computed tomography angiography, Three-dimensional visualization, Vascular diseases, Radiographic image interpretation

## Abstract

**Abstract:**

With the rapid advancement of multi-detector computed tomography (MDCT) and image post-processing technologies, CT angiography (CTA) has become a cornerstone in the diagnosis of vascular diseases. Despite its widespread use, conventional volume rendering (VR) is limited in depth perception, visualization of fine tissue textures, and differentiation of complex, overlapping anatomical structures. Cinematic rendering (CR), a next-generation visualization technique, utilizes a global illumination model to achieve photorealistic 3D reconstructions. This pictorial review is structured around representative CTA cases to illustrate the principles, clinical applications, and potential future directions of cinematic rendering in vascular imaging.

**Critical relevance statement:**

By reviewing the clinical applications of cinematic rendering in CT angiography, we summarize its advantages in visualizing complex vascular anatomy and explore its integration with emerging technologies, highlighting its role in optimizing preoperative planning and advancing precision medicine.

**Key Points:**

- Cinematic rendering employs global illumination and high dynamic range to surpass traditional volume rendering, enhancing anatomical realism for effective vascular diagnosis and lesion detection.
- Cinematic rendering transforms complex data into intuitive three-dimensional visualizations, reducing cognitive load for clinicians and facilitating both medical education and patient communication.
- Integration with artificial intelligence and mixed reality holds the potential to advance cinematic rendering from morphological displays to intelligent, interactive tools for preoperative planning.

**Graphical Abstract:**

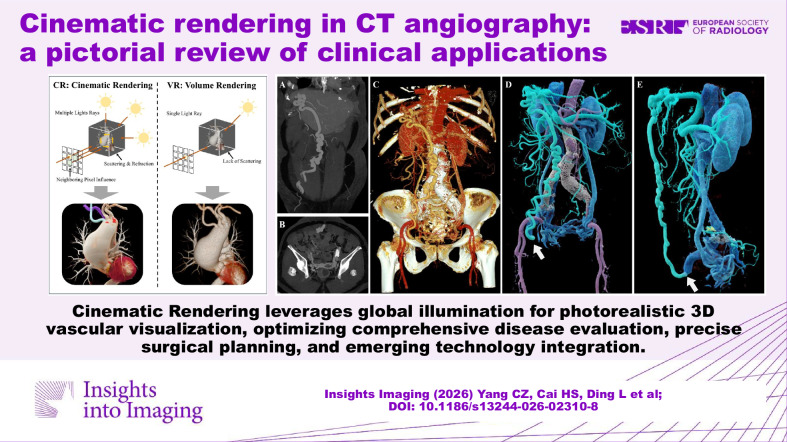

## Introduction

**Fig. 1:**
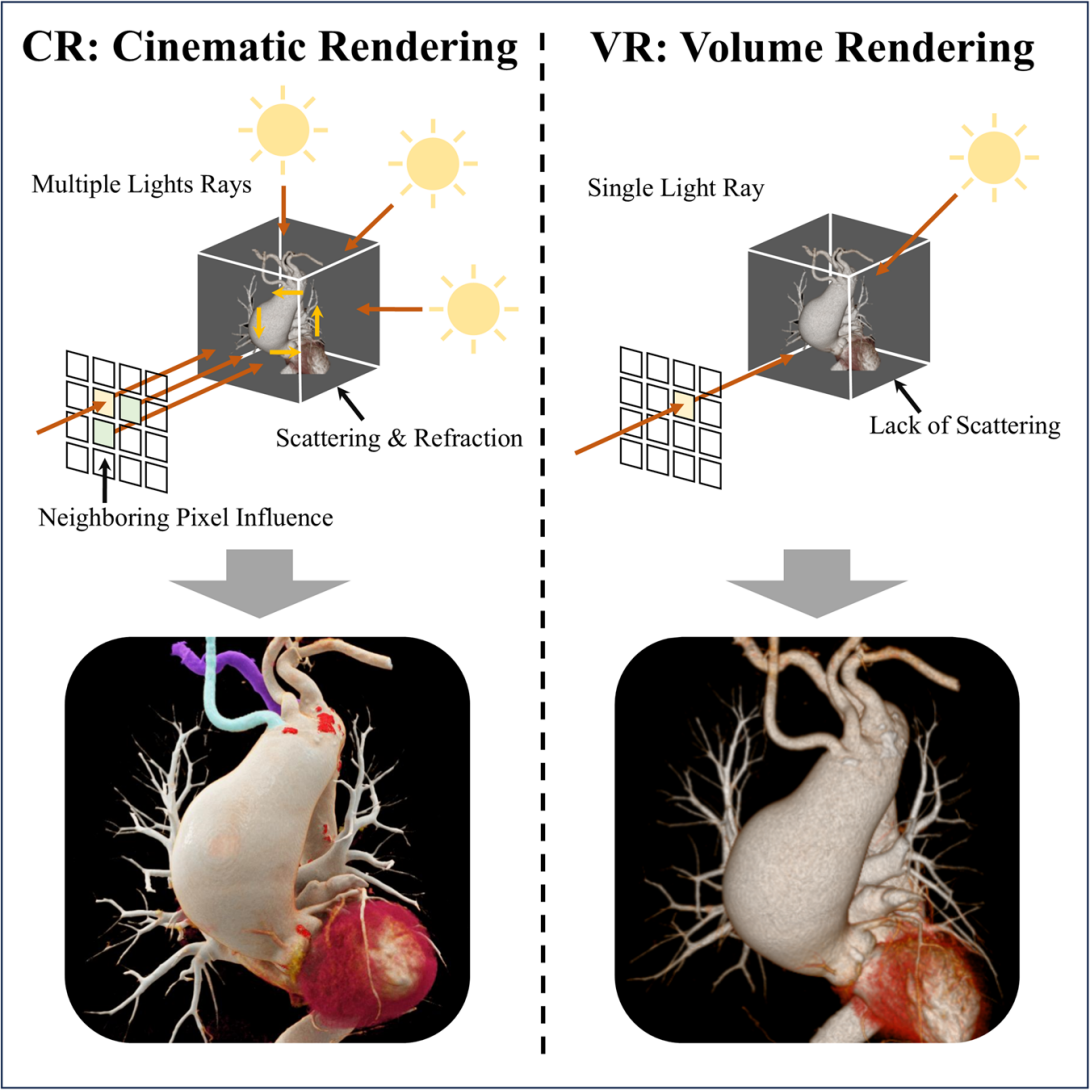
Schematic diagram illustrating the principles of cinematic rendering and volume rendering

Computed tomography angiography (CTA) has long been a cornerstone imaging modality for the pre- and postoperative assessment of vascular lesions, thanks to its rapid acquisition speed and high resolution. However, as clinical demand grows for photorealistic anatomical details and holistic stereoscopic visualization, the limitations of traditional three-dimensional (3D) visualization methods have been increasingly apparent. Volume rendering (VR) has traditionally dominated clinical practice due to its high computational efficiency and baseline diagnostic utility [1], but it is limited by a local lighting model based on Riemann integration. This approach does not account for scattered light and ambient occlusion, resulting in significant deficiencies in photorealism, depth perception, and the delineation of complex tissue boundaries in reconstructed images [2]. Consequently, while CTA remains the gold standard, its 3D visualization capabilities are restricted by traditional VR models, making it challenging to meet the spatial interpretation needs of complex vascular pathologies [3]. As a breakthrough solution, cinematic rendering (CR) technology introduces a cinematic-quality global illumination model [4]. Utilizing Monte Carlo path-tracing algorithms, CR not only calculates the primary light source but also tracks the scattering and refraction of light between adjacent pixels and tissues. This process imbues images with realistic, soft shadows and ambient occlusion effects, significantly enhancing their quality [5, 6]. The enhanced depth perception provided by this technology offers unparalleled advantages over traditional VR, particularly in restoring fine anatomical textures and resolving overlapping vascular structures. These capabilities establish CR as a core technology for next-generation volumetric reconstruction (Fig. 1 and Table 1).

**Table 1:** Comparison of technical principles and imaging characteristics between cinematic rendering and volume rendering

	Cinematic rendering (CR)	Volume rendering (VR)
Core algorithm	Path tracing: Employs Monte Carlo simulations to model multiple complex light paths.	Ray casting: Based on line-of-sight integration, primarily simulating the effects of a single light ray.
Lighting model	Global illumination model: Involves complex light-matter interactions.	Local illumination model: Involves simple light-matter interactions.
Image quality	Photorealistic quality; superior depth perception.	Synthetic appearance; limited depth perception.
Hardware dependency	High. Heavily relies on powerful GPUs for massive parallel computing.	Relatively low. Historically achievable on CPUs; efficiency is enhanced by GPU acceleration.

Currently, CR has demonstrated significant potential across multiple clinical domains, including cardiovascular imaging [7], the digestive system [8], skeletal pathologies [9], forensic medicine [10], and obstetrics [11]. Despite these preliminary explorations, the systematic evaluation of CR’s application and clinical value specifically within CTA remains relatively limited. Although current literature is largely confined to specific diseases or isolated anatomical structures, this pictorial review aims to provide a comprehensive, systemic overview of CR’s applications across various vascular beds. To demonstrate the visualization capabilities of CR, representative cases were retrospectively retrieved from our institutional Picture Archiving and Communication System (PACS). By synthesizing these diverse applications, we aim to offer new insights into how CR can serve as a unified platform that bridges morphological diagnosis, preoperative planning, and integration with emerging technologies.

## Clinical applications

This section presents the clinical applications of CR in vascular imaging, illustrated with retrospectively collected cases from our institution, following a structured rationale. We begin with arteriovenous malformations (AVMs) to highlight the exceptional ability of CR to resolve highly complex, tortuous spatial relationships. We then discuss its utility in overcoming low-contrast challenges in the venous system and its role in the comprehensive assessment of systemic arterial diseases. Finally, we address progress to the dynamic physiological challenges of thoracic cardiac imaging and conclude with the critical role of CR in perioperative structural evaluation.

### 3D visualization of AVMs

**Fig. 2:**
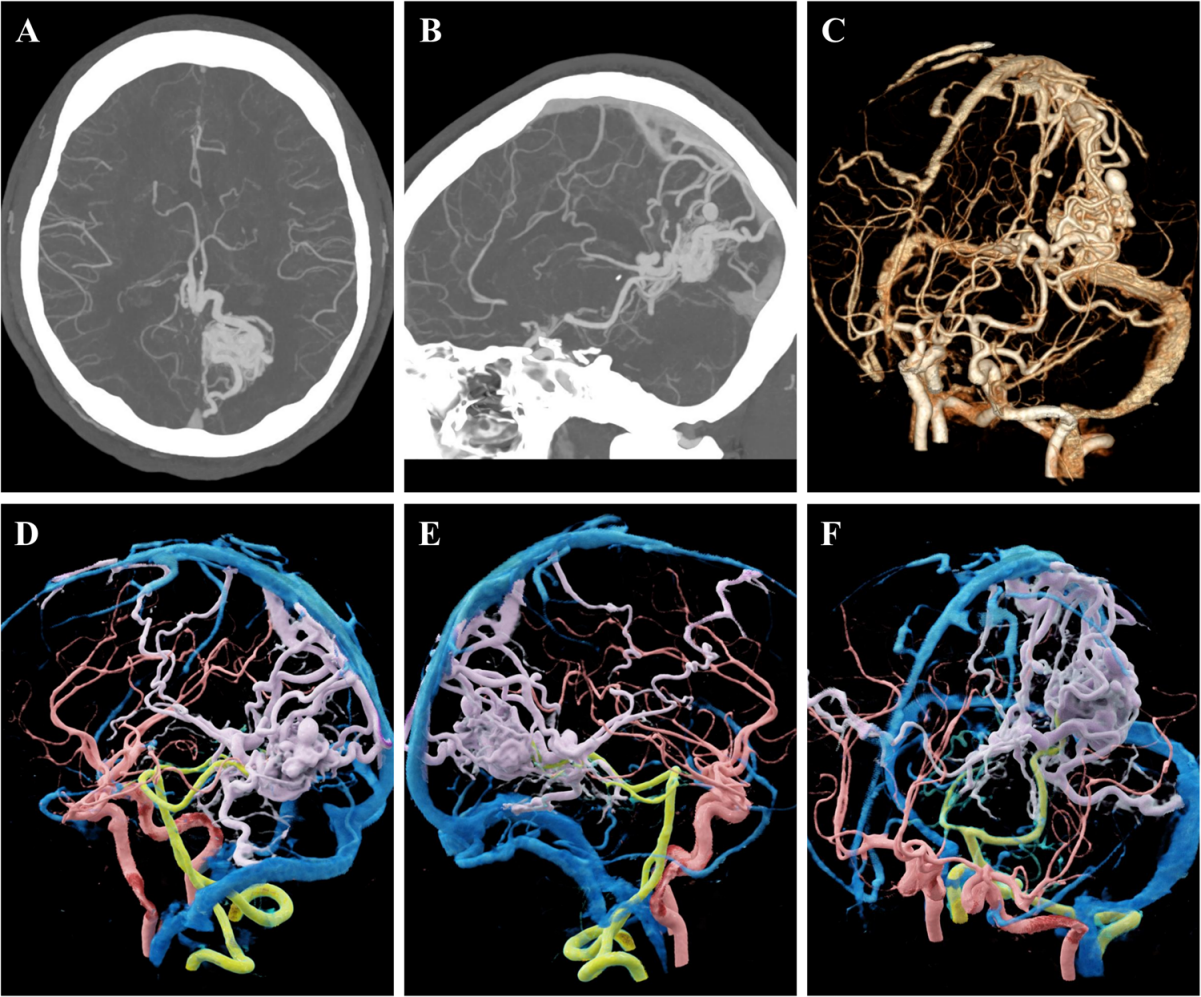
A 24-year-old male presenting with a 2-year history of recurrent headaches. **A**, **B** Maximum intensity projection (MIP) images reveal a tortuous vascular nidus in the left parieto-occipital region, highlighting feeding arteries and multiple draining veins. **C** VR reconstruction from CTA illustrates the overall contour of the vascular nidus. However, due to the malformed, tortuous, and intertwined nature of the vessels, the specific vascular course remains challenging to distinguish intuitively. **D**–**F** CR images of the same patient utilize high dynamic range lighting to clearly depict the stereoscopic structure of the vascular nidus. Through specific pseudo-color coding, the vascular anatomy is clearly delineated (red: internal carotid artery and anterior circulation; green: vertebral artery and posterior circulation; blue: veins; purple: abnormal vascular nidus). This distinct color coding elucidates the complex spatial relationships between normal and pathological vessels

**Fig. 3:**
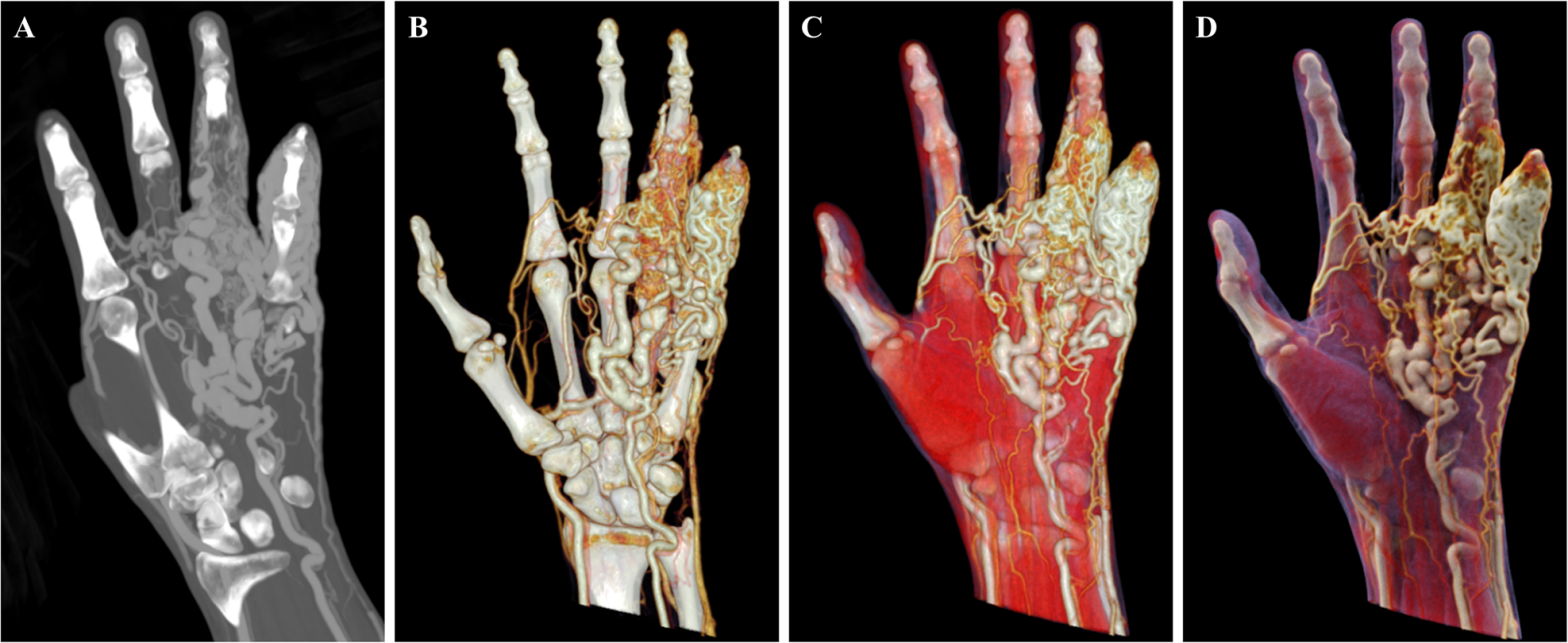
Hand arteriovenous malformation in a 39-year-old male. **A** The CTA-based MIP image reveals a tortuous vascular nidus involving the 3rd to 5th digits of the left hand; however, severe spatial overlap hinders accurate assessment. **B** VR reconstruction of the same patient demonstrates multiple tortuous pathological vessels. **C**, **D** CR images of the same patient, using varying threshold ranges, clearly delineate the spatial relationships among the vascular lesion, surrounding soft tissue, and bone, providing photorealistic 3D anatomical visualizations

AVMs are vascular anomalies characterized by abnormal direct shunts between arteries and veins, bypassing the capillary bed. Although AVMs may originate from various causes, acquired cases are more common than congenital ones [12]. Despite their low incidence, AVMs can cause significant hemodynamic alterations, leading to severe complications such as life-threatening hemorrhage and the failure of vital organs [13]. On CTA, AVMs are typically characterized by dilated feeding arteries, a tortuous and chaotic vascular nidus, and early opacification of draining veins (Figs. 2A, B, 3A) [14]. An accurate assessment of the origin, course, and spatial relationships of the tortuous vascular nidus is critical for effective preoperative planning.

While conventional 3D reconstruction techniques, such as maximum intensity projection (MIP) and VR, can delineate general contours, their lack of shadowing and depth perception often causes tortuous and entangled vessels to appear as a fused mass. This makes it difficult to distinguish the vascular course and intervascular relationships, resulting in suboptimal simulation (Figs. 2C, 3B). In contrast, CR leverages complex lighting models to vividly render intricate vascular structures (Figs. 2D–F, 3C, D). CR not only clearly delineates the stereoscopic architecture of the vascular nidus but also intuitively differentiates normal vessels from pathological ones using pseudo-color mapping. Furthermore, even in cases with severe lesion overlap, CR renders spatial relationships clearly visible through threshold adjustments. It provides a more intuitive perspective that closely mimics intraoperative findings, thereby facilitating clinical decision-making and guiding surgical or interventional management. In this context, CR vividly demonstrates the complex spatial entanglement and feeding-draining relationships of the AVM nidus, implying enhanced precision for preoperative navigation and risk stratification.

### 3D evaluation of venous system diseases

**Fig. 4:**
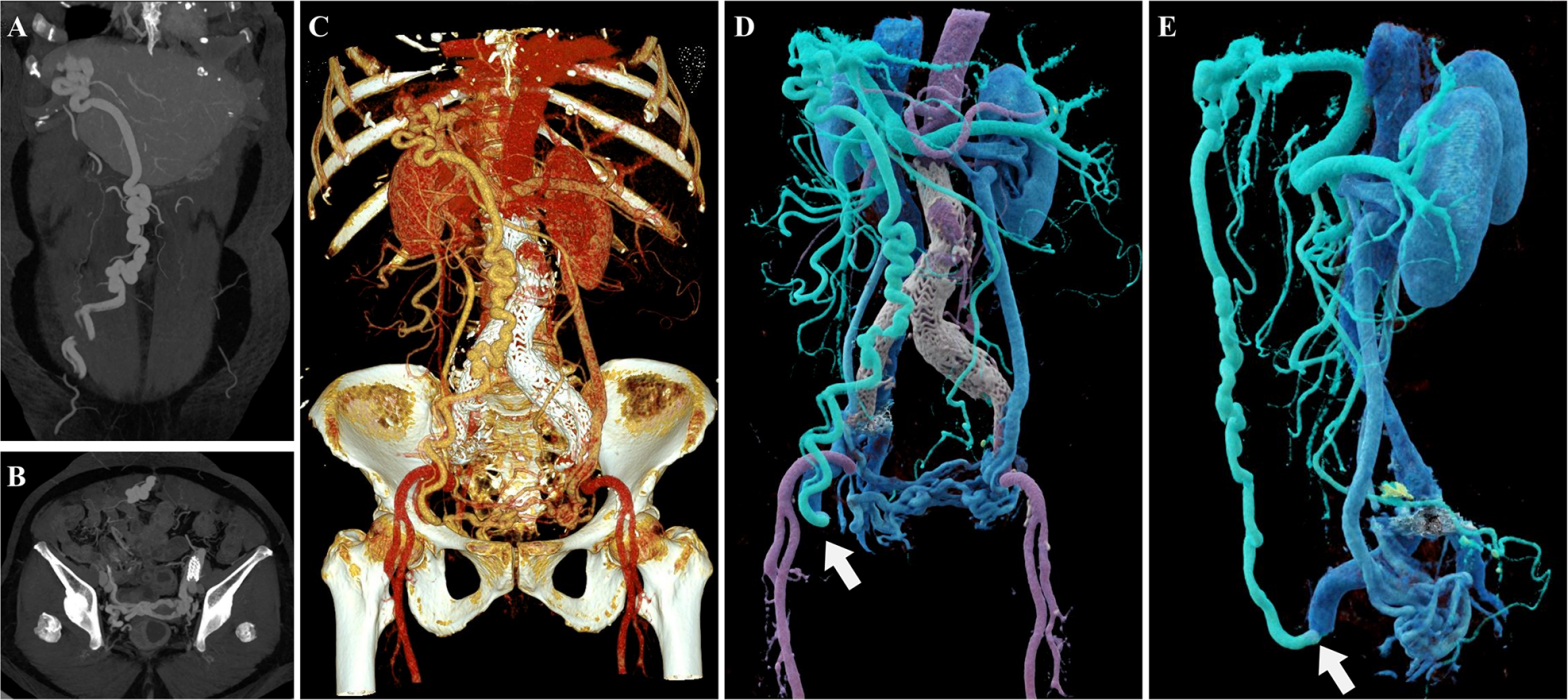
Abdominal imaging of a 78-year-old patient with portal hypertension. **A**, **B** Coronal and axial MIP images demonstrate the dilated portal vein and left ovarian vein, respectively. **C** A conventional VR image depicts the dilated venous lumen and its general spatial orientation; however, it offers limited visualization of fine pathological details. **D**, **E** CR images of the same patient clearly visualize post-aortic stenting changes. Furthermore, the photorealistic lighting rendering intuitively reveals an abnormal communication between the dilated portal vein and the right external iliac vein (white arrow), as well as significantly dilated ovarian varices. Color-coded rendering further elucidates the otherwise complex venous collateral relationships

**Fig. 5:**
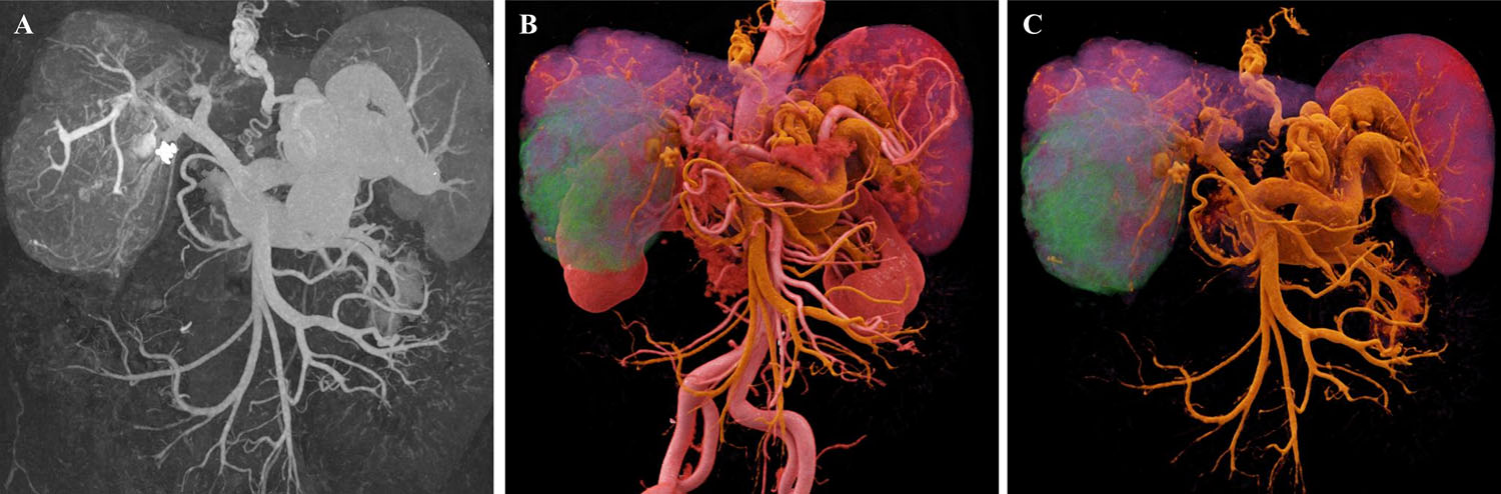
Images of a 62-year-old patient with liver cirrhosis and portal hypertension. **A** The CTA-based MIP image primarily displays dilated vascular shadows; however, due to vessel overlap, the image lacks spatial depth perception. **B**, **C** CR images of the same patient provide a stereoscopic view of the tortuous and dilated morphology of the portal vein, gastric fundal veins, and splenic vein. By masking the obscuring aortic structure, the anatomical details of the splenorenal shunt are intuitively revealed, providing a precise three-dimensional reference for the clinical evaluation of collateral circulation

Beyond its well-established role in arterial imaging, CR is irreplaceable for imaging the venous system, which is characterized by low intravascular pressure and reduced contrast agent concentration. Radiological assessment of the venous system is inherently challenging, primarily due to limited contrast opacification and the complex three-dimensional configuration of collateral pathways. Venous structures often remain obscured or poorly visualized in conventional post-processing reconstructions because of their lower contrast opacification compared to the arterial system and attenuation values similar to those of surrounding soft tissues (Figs. 4A–C, 5A). Furthermore, the full extent of complex collateral circulation is often difficult to intuitively visualize on 2D images [15]. CR leverages high dynamic range rendering and superior representation of low-contrast tissues to significantly enhance the visualization of venous vasculature. It enables clear and intuitive delineation of complex stereoscopic configurations—such as varices and collateral circulation—through advanced techniques like masking and color coding (Figs. 4D, E, 5B, C). In summary, CR enhances the visualization of low-contrast venous structures by improving depth cues and soft-tissue differentiation, enabling a more intuitive depiction of collateral circulation patterns.

### Comprehensive assessment of arterial thrombosis, arteriosclerosis, and associated complications

**Fig. 6:**
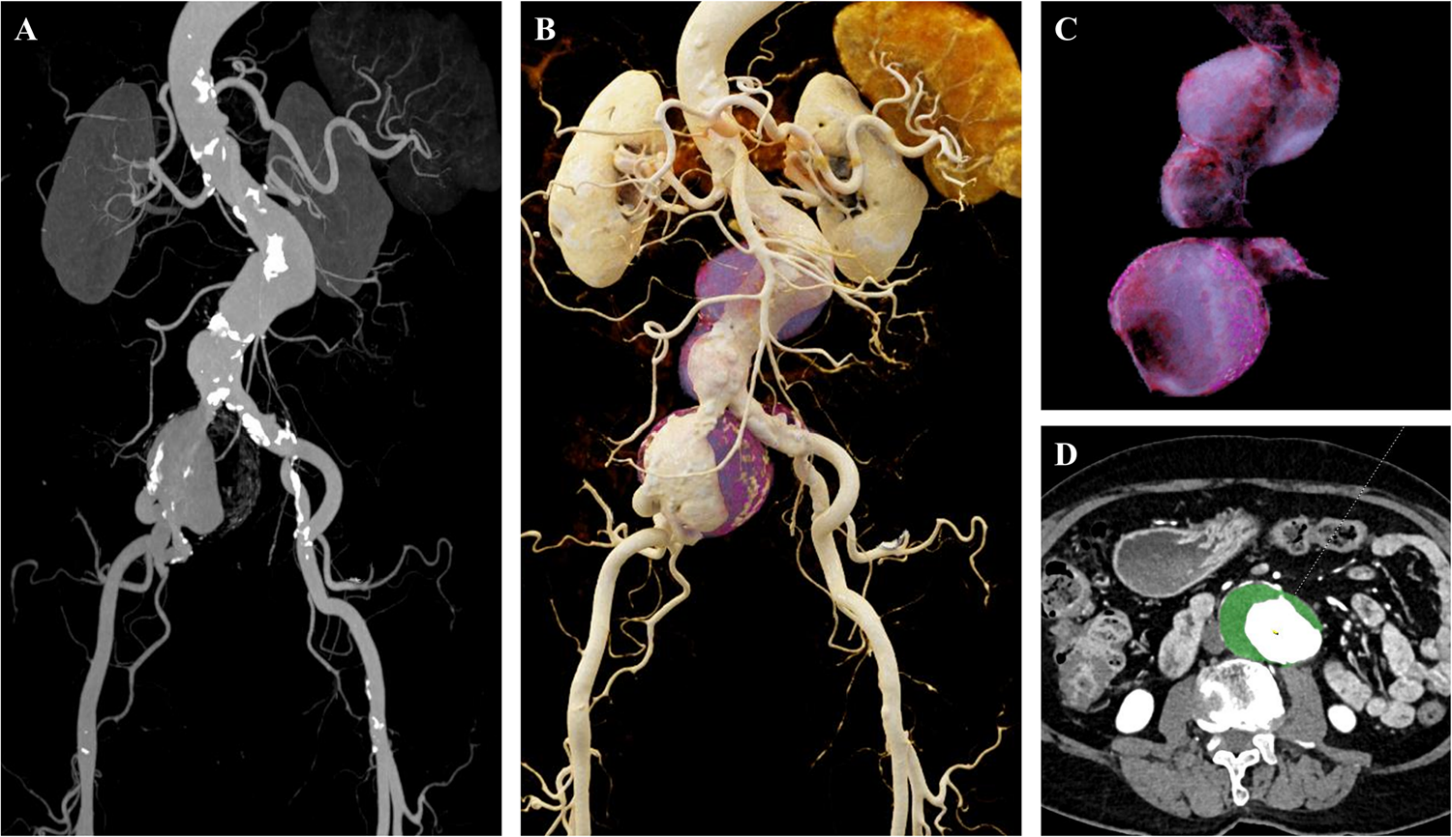
Abdominal aortic aneurysm in an 87-year-old patient. **A** The CTA-based MIP image reveals varying degrees of luminal dilation and stenosis in the distal abdominal aorta and right common iliac artery; however, the extent of the mural thrombus is challenging to intuitively visualize. **B** The CTA-based CR image vividly delineates the overall morphology of the aneurysm and the volumetric distribution of the mural thrombus (highlighted in purple). **C**, **D** CR reconstructions of the extracted mural thrombus (highlighted in purple in **C**, and green in **D**), segmented based on the region of interest, facilitate precise volumetric quantification and subsequent compositional analysis

**Fig. 7:**
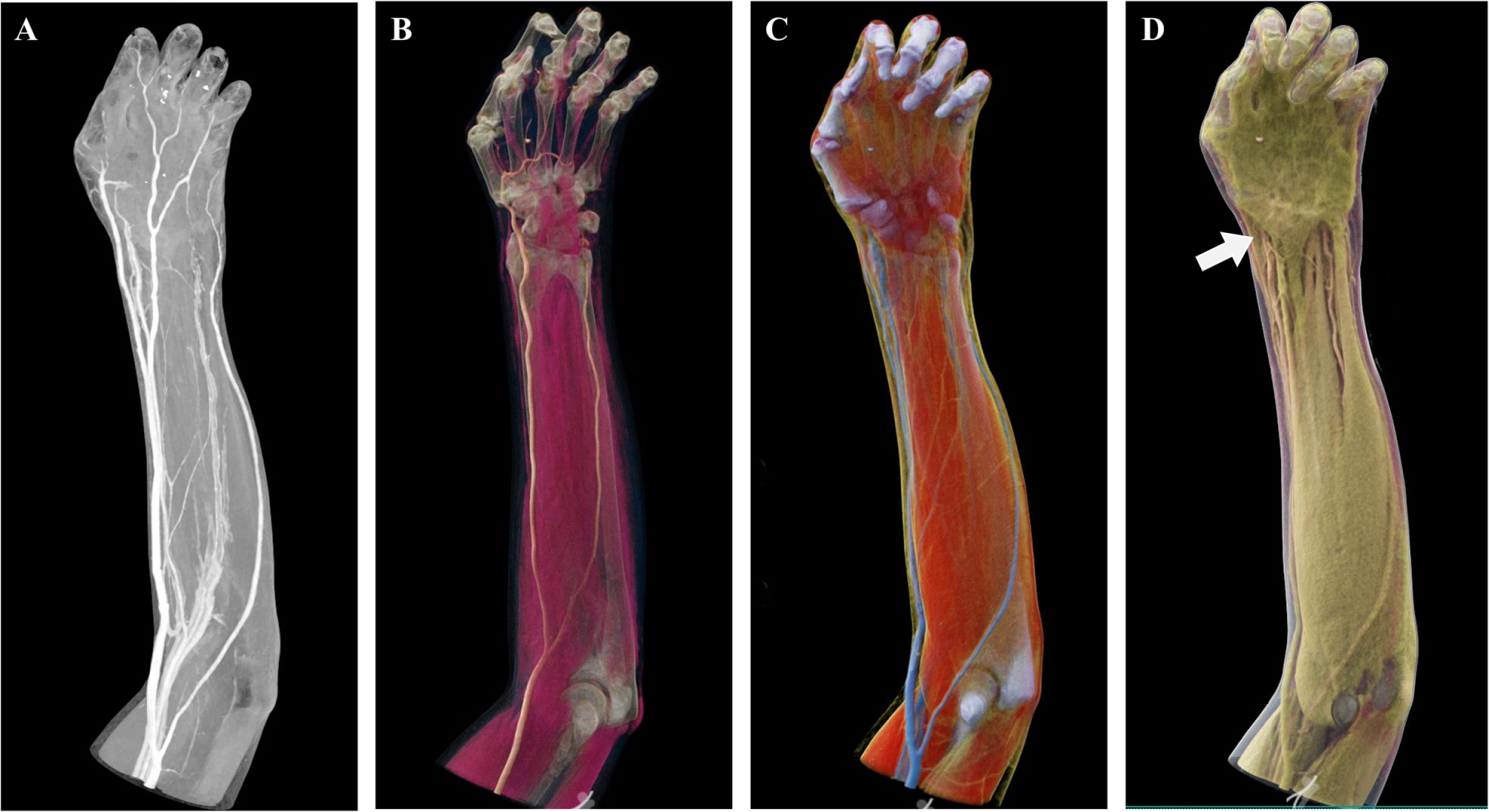
Palmar scar contracture with motion limitation in a 44-year-old male. **A** The CTA-based MIP image reveals occlusion of the distal right ulnar artery, its branches, and the distal branches of the deep palmar arch. **B**–**D** CR images, through adjustments in opacity and lighting, not only depict vascular pathologies but also realistically reconstruct the muscular anatomy. These images clearly demonstrate atrophy and collapse of the thenar muscles corresponding to the area of vascular occlusion, visually elucidating the causal relationship between vascular pathology and muscle atrophy; the arrow in **D** specifically indicates the significant atrophy and collapse of the thenar muscles. This approach achieves an integrated “vessel-end organ” comprehensive assessment

In addition to visualizing vascular morphology, the assessment of vessel wall pathologies and consequent end-organ changes has emerged as a new clinical focal point. Advancements in CTA scanning technology have extended the evaluation of vascular diseases beyond merely assessing luminal stenosis to emphasize pathological characteristics of the vessel wall, including mural thrombi, plaque composition, and associated complications [16, 17]. Traditional techniques such as MIP and VR primarily visualize hyperdense luminal structures, often overlooking low-density mural lesions and potentially underestimating the true vascular burden. These techniques also demonstrate limited capability in depicting the vessel wall and surrounding soft tissues (Figs. 6A, 7A). In contrast, CR excels in rendering the anatomical morphology of mural thrombi and perivascular tissues, enabling an integrated “lumen–wall–end organ” assessment (Figs. 6B, 7B–D). It should be noted that CR does not replace quantitative assessment based on axial images but provides an intuitive three-dimensional overview of the lumen–wall–end organ relationship. This capability holds significant clinical value for aneurysm volumetry, evaluating thrombus burden, and assessing related complications (Fig. 6C, D).

In the assessment of arterial disease, CR provides a comprehensive anatomical overview by facilitating the simultaneous visualization of the vascular lumen, vessel wall, and adjacent end-organ structures.

### Thoracic cardiac imaging and cinematic rendering motion

**Fig. 8:**
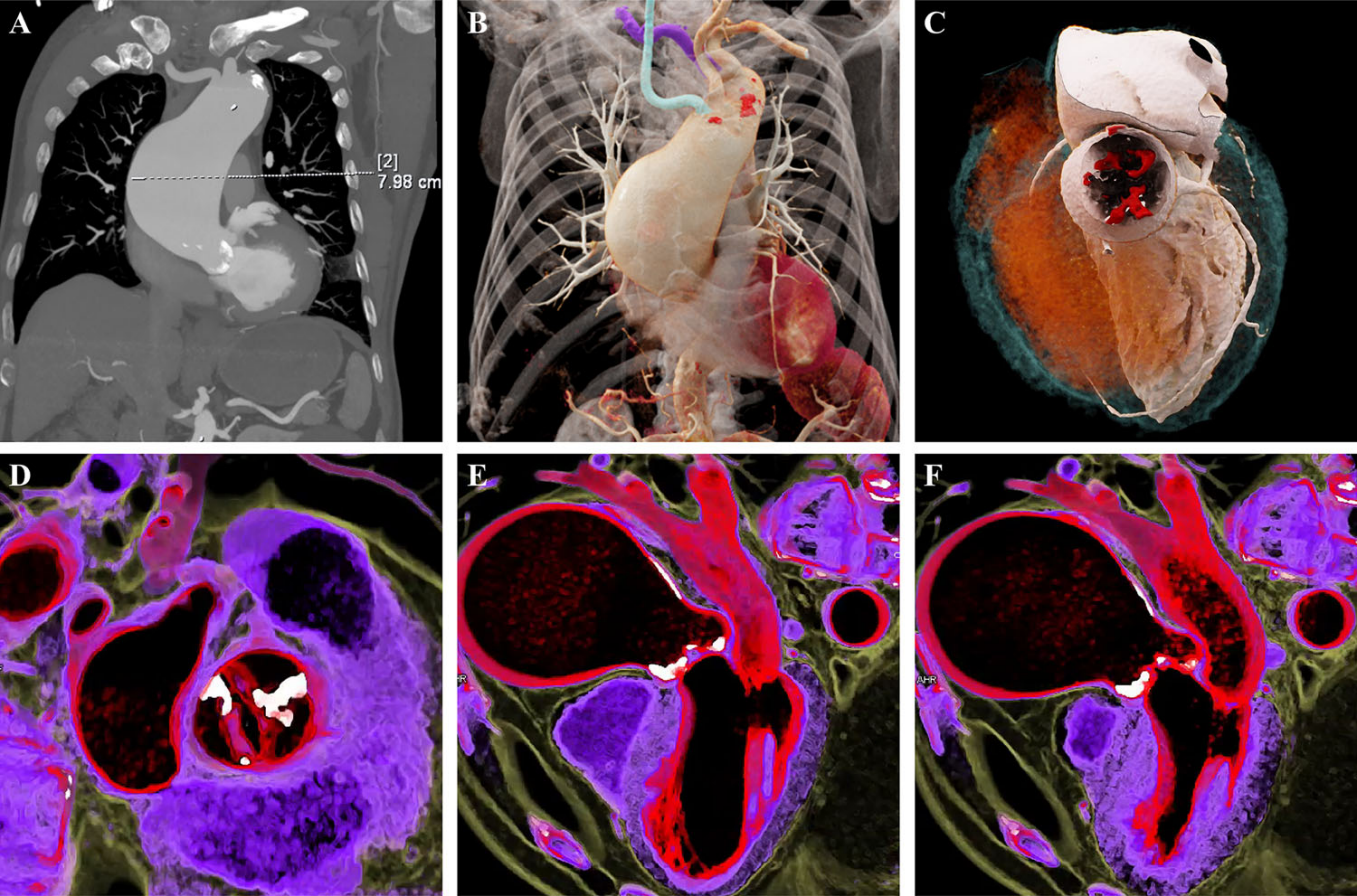
Chest CTA of a 78-year-old male with ascending aortic dilation and aortic valve calcification. **A** The MIP image shows a measurement indicating dilation of the ascending aorta to approximately 7.88 cm, with visible calcification of the aortic wall and valve. **B** The CR image intuitively visualizes the dilated morphology of the ascending aorta and mural calcified plaques (highlighted in red). It also clearly depicts anatomical variations, including an aberrant right subclavian artery (highlighted in purple), within the same field of view. **C** The CR image focuses on the aortic valve, stereoscopically displaying the distribution of calcified foci (red). **D**–**F** Dynamic cinematic rendering (4D-CR) of the aortic valve captures images at different phases of the cardiac cycle, dynamically demonstrating valve calcification and resultant motion restriction. Notably, during systole (**D**, **F**), valve opening is significantly restricted, providing an intuitive dynamic anatomical basis for valve replacement surgery

Distinct from the vascular imaging of other body regions discussed above, imaging of the thoracic heart and great vessels presents higher diagnostic demands due to the complex anatomical interplay between systemic and pulmonary circulations, as well as the inevitable physiological motion artifacts caused by cardiac and respiratory movements [18]. Although rapid scanning and electrocardiogram (ECG) gating techniques have mitigated some of these challenges, high-quality 3D reconstruction remains arguably the optimal method for image presentation [19, 20]. For structural heart diseases such as congenital heart anomalies and valvular pathologies, which result in complex morphological changes of the heart and great vessels, an intuitive understanding of abnormal 3D spatial relationships is paramount [21]. In this regard, CR technology not only provides a stereoscopic, “surgeon’s-eye” visualization of intricate congenital anomalies and aortic valve calcification, offering an intuitive reference for preoperative planning (Fig. 8B, C), but also integrates the temporal dimension through 4D-CR motion imaging. This technique captures valve morphology across different cardiac cycles; specifically, the dynamic visualization of restricted valve opening during systole effectively demonstrates motion impairment caused by calcification, providing intuitive dynamic anatomical evidence for valve replacement surgery (Fig. 8D–F). Furthermore, recent studies have utilized this technology to evaluate rare cardiovascular structural anomalies, such as complex coronary artery fistulas with giant aneurysms, demonstrating its exceptional capacity for anatomical delineation [22]. In these complex scenarios, CR significantly enhances the visualization of morphological characteristics and intricate spatial relationships, serving as a crucial visual adjunct for preoperative assessment. Currently, 4D-CR remains an emerging application, and its clinical role is complementary rather than substitutive.

In cardiac CTA, CR enables an integrated anatomical representation of complex cardiovascular structures, while emerging 4D-CR techniques offer a novel exploratory framework for visualizing motion-related abnormalities. Dynamic video files are available in the Supplementary Materials.

### Preoperative assessment: visualization of lesions and adjacent anatomy

**Fig. 9:**
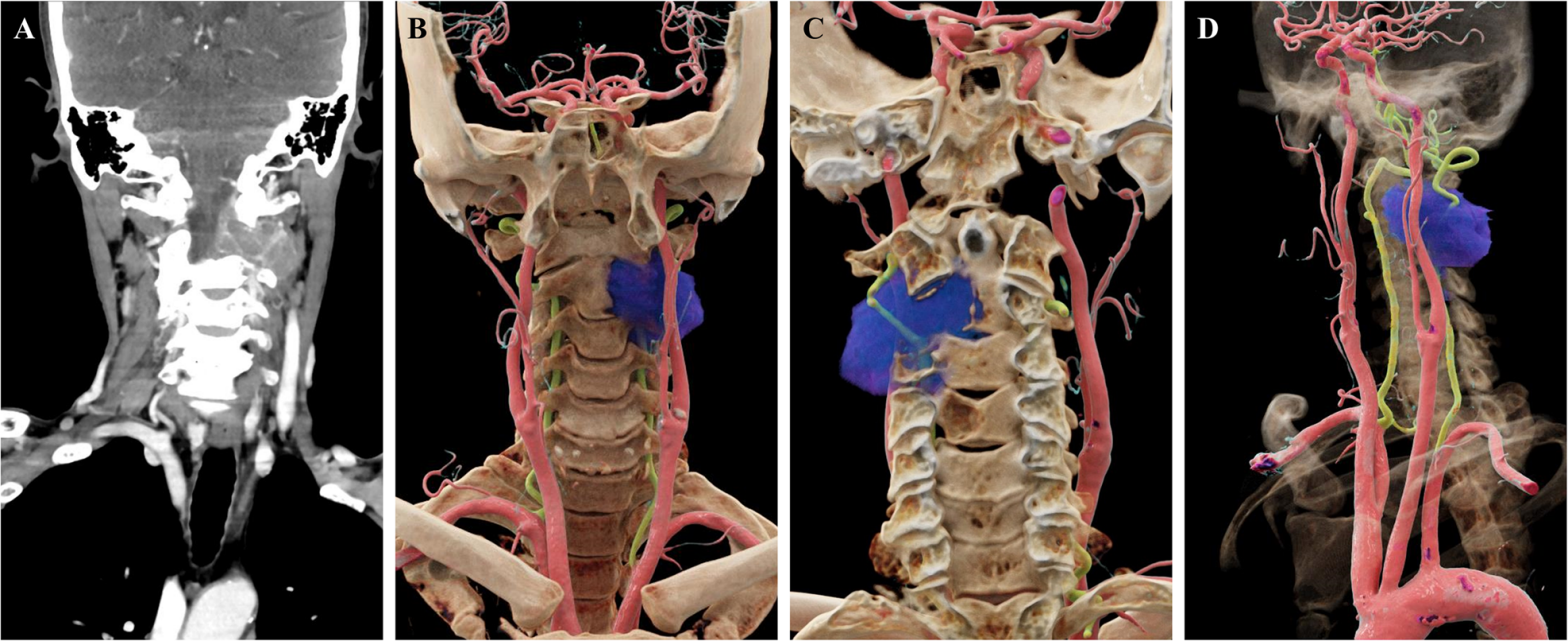
Images of a 68-year-old male with neck pain. **A** The coronal cervical CT image reveals bone destruction at the C2-C3 vertebral level, accompanied by the formation of a surrounding soft tissue mass. **B**–**D** Multi-angle CR images, with their superior depth perception capabilities, authentically depict the anatomical relationships among the tumor, bone, and blood vessels (blue: tumor; red: carotid artery; green: vertebral artery). The images clearly demonstrate that the V3 segment of the left vertebral artery is tightly encased by tumor tissue

Accurate preoperative anatomical assessment is pivotal for surgical success. CR technology, with its superior depth perception, serves as an important link between radiological imaging and surgical practice in preoperative tumor planning. Traditional 3D reconstructions often present with rigid textures, rendering the anatomical relationships between lesions and surrounding vessels as mere planar superimpositions that lack stereoscopic depth. Through meticulous post-processing, CR imaging not only facilitates visualization of tumor tissues with fine, anatomically realistic textures (Fig. 9B) but also clearly delineates the spatial relationships between tumors and adjacent vasculature, including abutment, encasement, and invasion (Fig. 9C, D). In the era of increasingly prevalent robot-assisted and minimally invasive surgeries, where the surgical field of view and operative angles are inherently restricted, such precise preoperative anatomical visualization is of paramount importance. CR imaging provides critical evidence for analyzing tumor resectability and formulating surgical strategies. Studies indicate that selective bone removal based on CR rendering can simulate the intraoperative perspective, assisting surgeons in avoiding high-risk vessels and determining the optimal surgical approach [23]. From a surgical planning perspective, CR contributes by clarifying the anatomical relationships between lesions and adjacent vascular structures, particularly regarding encasement, displacement, and invasion.

### Evaluation of postoperative vascular status and complications

**Fig. 10:**
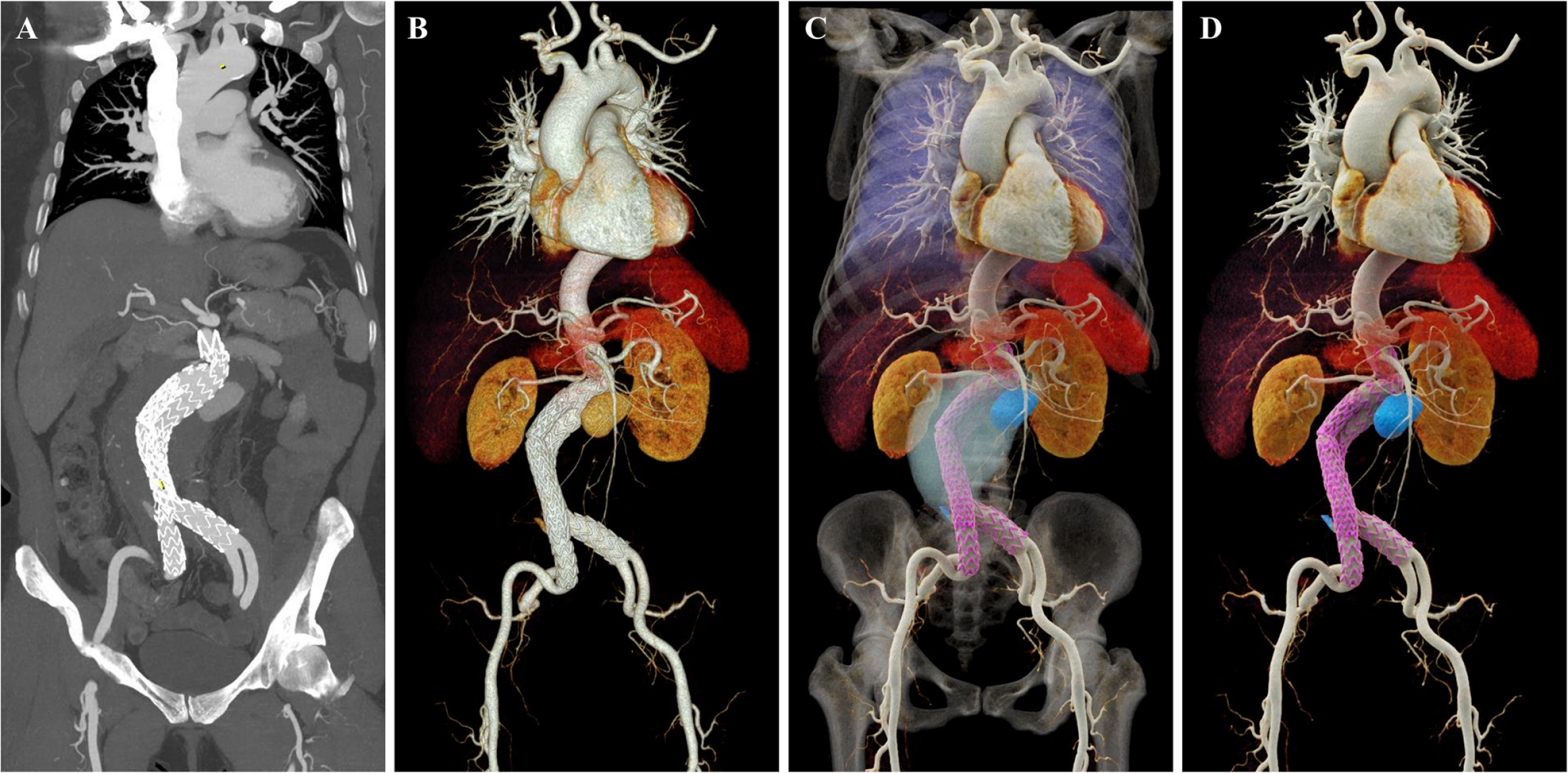
Follow-up images of a 61-year-old patient post-endovascular aneurysm repair (EVAR). **A** The CTA-based MIP image reveals stent shadows within the abdominal aorta and bilateral common iliac arteries, surrounded by slightly hypodense areas, with a visible localized endoleak. **B** The VR image visualizes only the contrast-enhanced portion of the endoleak, with non-enhanced regions poorly displayed. **C**, **D** CR images clearly depict the vessel wall and the mesh configuration of the metallic stent (purple). Utilizing high dynamic range rendering, these images distinctively differentiate between the surrounding chronic thrombus/organized vessel wall (light blue) and newly formed contrast-enhanced endoleak components (blue), precisely delineating the extent and source of the endoleak

**Fig. 11:**
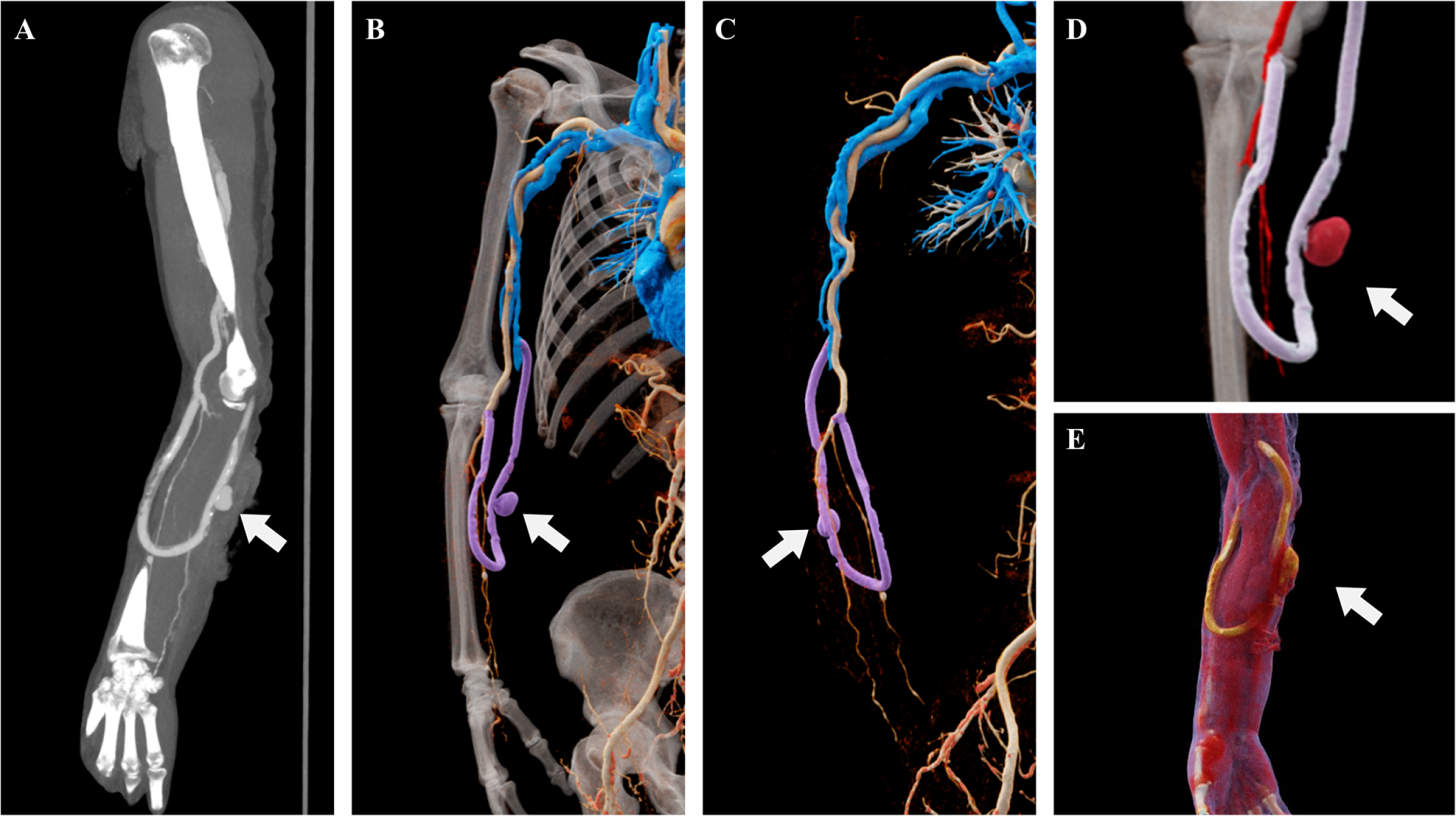
Follow-up images of a 65-year-old male after prosthetic arteriovenous fistula creation. **A** The CTA-based MIP image reveals an abnormal encapsulated hyperdense shadow surrounding the prosthetic graft, suggestive of hemorrhage (white arrow). **B**–**D** CR images stereoscopically and intuitively depict the morphology of the rupture site and hematoma, using anatomical segmentation and color coding to differentiate the artery, vein, prosthetic graft, and contrast extravasation following the rupture (white arrows). **E** The CR image further demonstrates the spatial and stratified relationships between the lesion (white arrow) and surrounding tissue structures, providing a precise anatomical roadmap for surgical exploration and repair

Postoperative vascular evaluation is equally significant, primarily focusing on monitoring implant status and associated complications. CTA serves as a pivotal modality for this purpose, enabling the rapid acquisition of volumetric data covering the entire surgical field to facilitate detailed assessment of soft tissues, vasculature, and implants. Previous studies have demonstrated that the diagnostic sensitivity of CTA for vascular injuries exceeds 90% [24]. However, CT-based assessment remains challenging due to postoperative anatomical alterations and artifacts introduced by endovascular devices. Leveraging its superior spatial visualization capabilities and advanced lighting rendering algorithms, CR effectively mitigates the impact of artifacts, allowing for the rapid and precise evaluation of postoperative status. In particular, CR is invaluable for post-stenting assessment, as it intuitively visualizes stent positioning, morphology, and fractures. Furthermore, it clearly delineates severe complications such as endoleaks (Fig. 10C, D) and hemorrhage (Fig. 11), providing critical decision support for reoperation or emergency management. While CR does not eliminate metal-related artifacts, it improves the anatomical interpretability of endovascular devices and surrounding structures in the postoperative setting, thereby facilitating the assessment of implant-related changes and complications.

In summary, across these clinical scenarios, the primary contribution of CR lies in enhancing anatomical understanding rather than replacing established CTA reconstruction techniques.

## Future perspectives and integration with emerging technologies

CR brings unprecedented photorealism and depth perception to medical imaging. By overcoming the limitations of traditional imaging in depicting textural details, CR is transitioning from a mere visualization tool to a source of 3D spatial data, enhancing clinical decision-making and interdisciplinary communication.

### CR and 3D printing

With advancements in materials science and precision medicine, 3D printing has emerged as a crucial tool for preoperative planning in complex vascular diseases [25]. Previous studies have noted that the fidelity of printed models is intrinsically dependent on the quality of the input data. Traditional 3D printing primarily relies on CT and VR imaging for data support. However, while these traditional images provide basic geometric information, they often lack the capacity to present detailed vascular surface textures, thereby limiting the authenticity of the printed models. In this context, CR provides a high-fidelity anatomical data foundation through voxel-level, photorealistic rendering. This data not only reconstructs anatomical textural details more accurately but also guides the selection of printing materials and parameter settings, enabling the construction of more precise 3D models [26, 27]. Although 3D printing still requires a trade-off between time and cost-effectiveness [28], CR-based 3D printed models have demonstrated superior value in improving surgical planning precision, enhancing spatial memory, and facilitating medical education and simulation training [29].

### CR in mixed reality and intraoperative navigation

CR offers significant advantages in providing depth perception and stereoscopic spatial relationship information, which are critical for optimizing surgical planning and intraoperative navigation. In traditional surgical navigation systems, surgeons often face limitations due to the surgical field of view and the need to frequently switch their gaze between the patient and remote monitors. This interaction can disrupt hand-eye coordination and increase the intraoperative operational burden. Unlike Virtual Reality, Mixed Reality (MR) addresses this issue by using head-mounted displays to superimpose holographic 3D images directly onto the real world [30], which has been well-demonstrated in complex craniomaxillofacial surgeries [31]. The introduction of CR further enhances the clinical potential of MR. Compared to conventional volume rendering, CR provides more realistic spatial positioning and tissue hierarchy presentation. This high-fidelity rendering allows surgeons to clearly perceive subcutaneous blood vessels and their spatial relationships while directly viewing the patient, enabling real-time manipulation of holographic images via gestures or interactive interfaces in various oncological and neurosurgical fields [32, 33]. Furthermore, preliminary studies suggest that CR-driven MR navigation optimizes surgical workflows for open vascular procedures [34]. It also significantly reduces unnecessary exploratory maneuvers during perforator mapping [35], thereby minimizing the risk of intraoperative complications and enhancing overall surgical efficiency during complex aneurysm planning [36].

### CR and artificial intelligence

Artificial intelligence is fundamentally transforming the paradigm of medical image analysis, yet its performance is highly dependent on the quality and information density of the training data. Traditional CT images and VR often lack or lose textural information, which limits the ability of AI models in fine recognition and prediction tasks. Conversely, CR introduces lighting models that closely mimic physical reality, preserving minute textural features and complex spatial information. This high-quality data input holds the potential to significantly improve the AI models’ performance in detecting and predicting minute lesions. It is crucial to ensure “anatomical authenticity” and avoid introducing non-biological artifacts, which remain essential prerequisites for applying CR data to AI research [37]. With these considerations in mind, the synergy between CR and AI is expected to enhance the detection capabilities and predictive performance of AI models [38].

### CR in multidisciplinary collaboration and doctor-patient communication

When dealing with complex anatomical structures, traditional 2D grayscale CT and VR images often appear abstract and obscure, making it difficult to intuitively reflect stereoscopic spatial relationships. This can impose a high cognitive load on non-radiologists. In contrast, cinematic rendering (CR) technology transforms tomographic data into intuitive, lifelike volumetric images [39]. This assists surgeons of varying experience levels and medical trainees in clearly identifying minute vascular lesions and spatial relationships [40, 41]. Furthermore, it translates professional imaging data into accessible visual information, significantly reducing the difficulty for patients to understand their condition and surgical risks [42], thereby facilitating routine clinical implementation and shared decision-making [43]. Ultimately, by breaking down disciplinary barriers, CR reduces communication costs and promotes deep collaboration within multidisciplinary teams, while powerfully driving the paradigm shift toward “Patient-Participatory Medicine” and fostering a harmonious doctor-patient relationship.

Across these domains, CR emerges as a multifunctional platform that bridges imaging, preoperative planning, intraoperative guidance, AI-based analysis, and patient communication. Continued technical development and rigorous clinical validation are essential to fully realize its potential in precision medicine, including improving surgical outcomes, enhancing AI model performance, and facilitating patient understanding.

## Practical considerations and limitations

Despite its clear advantages in CTA, several practical and technical considerations must be addressed before CR can be widely adopted in clinical practice. First, CR relies on complex Monte Carlo path-tracing algorithms, which require high-performance workstations equipped with advanced GPUs and proprietary software. The associated costs for hardware acquisition and software licenses may restrict access, particularly in smaller hospitals or primary care institutions. Second, post-processing is initially more demanding than conventional VR, as technologists and radiologists must learn to optimize transfer functions and virtual lighting. However, once standardized templates for specific vascular regions are established, routine CR generation can typically be completed within a few minutes per case by trained personnel. Third, the quality of CR images depends heavily on the quality of the source CT data, underscoring the need for meticulous acquisition protocols. Finally, like other 3D reconstruction methods, CR carries a potential risk of obscuring or overlooking lesions. Adjusting opacity and lighting to highlight one anatomical layer may inadvertently mask findings in adjacent structures or small branches. As illustrated in Fig. 7, although CR produces highly photorealistic renderings of vessel walls, it may not provide the fully transparent, uninterrupted overview of the entire vascular tree afforded by conventional MIP. Therefore, CR should always be interpreted in conjunction with standard CT reconstructions to ensure a comprehensive assessment. Given that most current evidence remains qualitative, further studies are needed to rigorously evaluate its impact on diagnostic accuracy and patient outcomes.

## Conclusion

CR provides high-fidelity, photorealistic three-dimensional visualization in CTA, significantly enhancing the depiction of vascular morphology, pathologies of the vessel wall, and dynamic cardiac motion. Compared to conventional VR and MIP, CR improves depth perception, tissue texture, and the spatial delineation of complex vascular structures. This supports accurate preoperative planning, postoperative assessment, and comprehensive evaluation of vascular disease. Moreover, CR datasets can also be integrated with artificial intelligence, mixed reality, and 3D printing to facilitate advanced image analysis, intraoperative guidance, and patient-specific modeling. These capabilities establish CR as a versatile platform for precision medicine. However, further clinical validation and integration into routine workflows are necessary to fully realize its potential in improving diagnostic accuracy, surgical outcomes, and multidisciplinary decision-making.

## Supplementary information

Supplementary Material 1

Supplementary Material 2

## Data Availability

The datasets generated and analyzed during the current study are not publicly available due to sensitive patient information. However, they are available from the corresponding author upon reasonable request.

## Abbreviations

3D: Three-dimensional
AI: Artificial intelligence
AVM: Arteriovenous malformations
CR: Cinematic rendering
CTA: Computed tomography angiography
MIP: Maximum intensity projection
MR: Mixed reality
VR: Volume rendering
